# Supplementary material for: Two Years of Viral Metagenomics in a Tertiary Diagnostics Unit: Evaluation of the First 105 Cases
Source: Genes (Basel). 2019 Aug 29;10(9):661. doi: 10.3390/genes10090661 (PMC6770117; doi:10.3390/genes10090661)
Supplement: Supplementary file 1 [file genes-10-00661-s001.zip › Suppl_Figure_S2.html]

Javascript must be enabled to view this page.

magnitude
score


virus reported
virus found

 61
 76

 .491803278688525
 .565789473684211

 11
 16

 1
 1

 1
 2

 1
 1

 10
 14

 1
 1

 4
 4

 0
 0

 4
 4

 0
 0

 1
 1

 0
 0

 1
 1

 0
 0

 11
 12

 0
 0

 1
 1

 0
 0

 1
 2

 0
 0

 7
 7

 0
 0

 1
 1

 0
 0

 1
 1

 0
 0

 2
 2

 1
 1

 2
 2

 1
 1

 9
 14

 1
 1

 2
 3

 1
 1

 1
 1

 1
 1

 1
 1

 1
 1

 1
 1

 1
 1

 4
 6

 1
 1

 0
 2

 0
 1

 1
 1

 1
 1

 1
 1

 1
 1

 2
 2

 0
 0

 2
 2

 0
 0

 3
 4

 1
 1

 1
 1

 1
 1

 1
 1

 1
 1

 1
 1

 1
 1

 0
 1

 0
 1

 1
 1

 0
 0

 1
 1

 0
 0

 7
 7

 0
 0

 1
 1

 0
 0

 1
 1

 0
 0

 3
 3

 0
 0

 2
 2

 0
 0

 4
 6

 1
 1

 4
 4

 1
 1

 0
 1

 0
 1

 0
 1

 0
 1

 5
 5

 0
 0

 5
 5

 0
 0

 0
 1

 0
 0

 0
 1

 0
 0
